# Achieving High-Energy-Density Graphene/Single-Walled Carbon Nanotube Lithium-Ion Capacitors from Organic-Based Electrolytes

**DOI:** 10.3390/nano14010045

**Published:** 2023-12-22

**Authors:** Hang Yin, Jie Tang, Kun Zhang, Shiqi Lin, Guangxu Xu, Lu-Chang Qin

**Affiliations:** 1 National Institute for Materials Science, 1-2-1 Sengen, Tsukuba 305-0047, Ibaraki, Japan; yin.hang@nims.go.jp (H.Y.); zhang.kun@nims.go.jp (K.Z.); lin.shiqi@nims.go.jp (S.L.); xu.guangxu@nims.go.jp (G.X.); 2 Graduate School of Pure and Applied Sciences, University of Tsukuba, 1-1-1 Tennodai, Tsukuba 305-0006, Ibaraki, Japan; 3 Department of Physics and Astronomy, University of North Carolina at Chapel Hill, Chapel Hill, NC 27599-3255, USA; lcqin@email.unc.edu

**Keywords:** lithium-ion capacitor, graphene, asymmetric capacitor, LiBETI, three-dimensional structure

## Abstract

Developing electrode materials with high voltage and high specific capacity has always been an important strategy for increasing the energy density of lithium-ion capacitors (LICs). However, organic-based electrolytes with lithium salts limit their potential for application in LICs to voltages below 3.8 V in terms of polarization reactions. In this work, we introduce Li[N(C_2_F_5_SO_2_)_2_] (lithium Bis (pentafluoroethanesulfonyl)imide or LiBETI), an electrolyte with high conductivity and superior electrochemical and mechanical stability, to construct a three-electrode LIC system. After graphite anode pre-lithiation, the anode potential was stabilized in the three-electrode LIC system, and a stable solid electrolyte interface (SEI) film formed on the anode surface as expected. Meanwhile, the LIC device using LiBETI as the electrolyte, and a self-synthesized graphene/single-walled carbon nanotube (SWCNT) composite as the cathode, showed a high voltage window, allowing the LIC to achieve an operating voltage of 4.5 V. As a result, the LIC device has a high energy density of up to 182 Wh kg^−1^ and a 2678 W kg^−1^ power density at 4.5 V. At a current density of 2 A g^−1^, the capacity retention rate is 72.7% after 10,000 cycles.

## 1. Introduction

Electrochemical energy storage devices, such as lithium-ion batteries (LIBs) and electric double-layer capacitors (EDLCs), have made great strides in the past decade [1,2,3]. Commercial LIBs can store energy densities of 150–200 Wh kg^−1^ [4,5]. However, their power output (<1 kW kg ^1^) and lifetime (<10^−3^ times) are not as satisfactory as expected [6,7]. Conversely, EDLCs can achieve power densities and lifetimes of >5000 W kg^−1^ and >100,000 cycles, respectively [8,9]. However, the energy densities in EDLC with organic electrolytes are less than 10 Wh kg^−1^ [10,11,12]. Therefore, combining the high energy density of batteries and the high power density and cycle life of EDLCs, hybrid lithium-ion capacitors (LIC) have received great attention in recent years [13,14,15]. 

In recent years, many efforts have been made to obtain higher energy-density LIC devices for fabrication, cathode and anode electrode material synthesis and electrolyte synthesis. Capacitor-type cathode materials are mainly composed of carbon-based materials such as activated carbon [16], template carbon [17], graphene [18], and their composites. Graphene-based carbon materials are widely used in LIC cathodes due to their unique two-dimensional structure, porous structure, superior electrical conductivity, and much larger specific capacitance. However, Graphene sheets can severely aggregate and reaccumulate, decreasing capacity after several charge/discharge cycles. There have been many efforts to solve this issue, and one effective method is to form 3D nanostructures by using carbon nanotubes as spacers in the graphene sheet layers. This structure can provide enough space for charge storage and numerous paths for ion transport [19,20]. For example, Xiao and co-workers prepared graphene/CNT 3D composite structural materials using microwave irradiation. When used as the cathode and anode of LIC, graphene/CNT showed a high energy density of 232.6 Wh kg^−1^ at 226.0 W kg^−1^ [21]. 

The other factor that affects energy density and power density is the mismatch in kinetics between the cathode and anode. Generally, the anode kinetics based on Li-ion intercalation/ deintercalation is much slower than the cathode based on the EDLC model, leading to lower energy densities at relatively high power densities [22]. This means that when the power density is high, the energy density is low. Therefore, finding an electrolyte that matches the kinetics of the cathode and anode is vital to address the high energy and power density demands of LICs. Li[N(C_2_F_5_SO_2_)_2_] (LiBETI) has superior ionic conductivity and capacity retention compared to conventional LiPF_6_ used in lithium-ion batteries [23]. In an LIC, superior ionic conductivity provides the reaction kinetics, and LiBETI can also maintain the stability of the anode after pre-lithiation. So far, few reports have investigated using LiBETI as an electrolyte for lithium-ion capacitors.

In this work, a 3D porous structure is achieved using SWCNTs as spacers inserted into the graphene sheets. It can effectively prevent the stacking of graphene sheets. The synthesized graphene/SWCNT composite is used as the LIC cathode, and the pre-lithiated graphite is used as the anode to assemble a three-electrode system LIC device. The results show that using LiBETI as the electrolyte, the LIC devices have better conductivity and higher specific capacity than the general lithium salt electrolyte, such as lithium hexafluorophosphate (LiPF_6_) and lithium difluorosulfimide (F_2_LiNO_4_S_2_ or LiFSI). Analyzing the anode potential in the three-electrode system shows that the anode potential remains almost constant at all charge and discharge voltages (at 3.8–4.5 V). It indicates that LiBETI can make the anode form a stable SEI film. As will be described in detail in this report, the hybrid LIC device has a high energy density of up to 182 Wh kg^−1^ and a 2678 W kg^−1^ power density at 4.5 V. At a current density of 2A g^−1^, the capacity retention rate is 72.7% after 10,000 cycles. 

## 2. Materials and Methods

### 2.1. Materials

#### 2.1.1. Reagents

Graphite flakes with a size of about 10 μm were purchased from NSC, Japan. Hydrazine hydrate was purchased from Wako Pure Chemical Industries, Tokyo, Japan. The single-walled carbon nanotube was purchased from Nippon Zeon Co., Ltd. (Tokyo, Japan). The anode electrode material P_5_B was purchased from Nippon Carbon Co., Ltd. (Tokyo, Japan) The electrode film conductive agent, Ketjen Black, was purchased from Nippon Ketjen Co., Ltd. (Tokyo, Japan) The electrode film binders polyvinylidene difluoride (PVDF) and the electrolytes lithium salt used lithium hexafluorophosphate (LiPF_6_), lithium difluorosulfimide (LiFSI), and LiBETI from Tokyo Chemical Industry Co., Ltd. (Tokyo, Japan) The solvent ethylene carbonate (EC) and diethyl carbonate (DEC) were from Tokyo Chemical Industry Co., Ltd. 

#### 2.1.2. Synthesis of the Graphene/CNT Composite

Graphene oxide (GO) was synthesized from graphite flakes using a modified Hummers method. Details of the method have been reported in our previous publications [24]. 5 g of natural graphite, 3.75 g of NaNO_3_, and 310.5 g of H_2_SO_4_ were placed in a beaker and stirred in an ice bath at 0 °C for 30 min. Under stirring, 22.5 g of KMnO_4_ was slowly added to the above solution, and the temperature was kept below 10 °C. The resulting mixture was then stirred at room temperature for 2 days, after which 1 L of concentrated H_2_SO_4_ was added dropwise over a 1 h period. The mixture was stirred until it cooled down. After that, 150 g of H_2_O_2_ (30%, Aldrich, Tokyo, Japan) was added to the mixture to change the color of the suspension from reddish brown to yellow. Specifically, after obtaining the GO solution, the GO suspension was diluted with 0.1 mol L^−1^ HCl, centrifuged five times, diluted with deionized water, and continuously centrifuged (30,000 rpm/min) until the pH of the supernatant reached 7 to remove impurities. Graphene/SWCNT composites were prepared by mixing SWCNT and GO in the ultrasonic rod. The SWCNT was well dispersed in GO suspension and reduced by adding hydrazine hydrate for 24 h. The mixture was then filtered to obtain graphene/SWCNT slurry. The mixture suspension was then filtered to obtain an SG slurry. Subsequently, the slurry was washed with distilled water and vacuum-dried to obtain the SG composite as dry powder. Pure graphene without adding carbon tubes was also produced using the same method.

### 2.2. Material Characterization

The morphology and structure of the synthesized graphene/SWCNT were characterized using scanning electron microscopy (SEM, JSM-7001F, JEOL, Tokyo, Japan) and transmission electron microscopy (TEM, JEM-2100, JEOL, Tokyo, Japan). In addition, the structure was investigated with powder X-ray diffraction (XRD, Rigaku SmartLab (Kyoto, Japan), Cu-Kα radiation, λ = 1.5418 Å). The functional groups on GO and graphene were characterized by X-ray photoelectron spectroscopy (XPS, ULVAC-PHI Quantera SXM, Kanagawa, Japan) and Fourier transform infrared spectroscopy (FTIR, Shimadzu, IRTracer-100, Kyoto, Japan). Raman spectroscopy (RAMAN-11 with a 532 nm laser source, Nanophoton) was used to analyze the D-band and G-band peaks associated with the graphite structure in the composites. Nitrogen adsorption–desorption data (Quantachrome Autosorb iQ) were collected to calculate the specific surface area using the Brunauer–Emmett–Taylor (BET) method and density Functional Theory (DFT) calculations to obtain the pore size distribution.

### 2.3. Electrochemical Measurement

The three-electrode system’s working electrode (cathode) is graphene/SWCNT, the counter electrode (anode) is graphite, and the reference electrode is lithium foil. All the electrodes were prepared using the traditional slurry electrode fabrication route and dried in a vacuum. The cathode and anode were made with 85 wt% active materials, 10 wt% of PVDF, and 5% Ketjen Black. The anode electrode material was coated on a porous copper foil and the cathode electrode material was coated on an aluminum foil. The lithium foil was fixed to the copper foil as a reference electrode by pressing it with a roller. Next, 1 mol/L electrolyte was prepared with LiPF_6_/LiFSI/LiBETI as lithium salt solute and EC and DEC (EC/DEC 1:1, *v/v*) mixed solution as solvent. To explore the cathode capacity, a symmetrical EDLC pouch cell was assembled using the above electrolyte and a 25-micron cellulose separator. Pre-lithiated graphite anode: the graphite electrode was connected to the lithium electrode to form a half cell, which was discharged to 0.08 V at 20 mA g^−1^ and then kept at the potential for 30 h to reach the lithiated state. To prevent over-discharge of the anode, the LIC was tested at a potential of 2.2~4.5 V. Electrochemical impedance spectroscopy (EIS) measurement was also performed over a frequency range from 20 kHz to 0.2 Hz. All electrochemical tests were performed using an electrochemical workstation (Biologic VSP-300).

The specific capacitance, energy density, and power density of the material were calculated from the constant current charge/discharge curve at different current densities. Electrode mass calculates the total mass of the cathode and anode. The calculation formula is as follows:

(1) $$C=\frac{I\times t}{m\times ∆V}$$

where *I* is the discharge current, *m* is the cathode’s and anode’s total active mass, *t* is the discharge time, and Δ*V* is the voltage difference between charge and discharge.

The formula for calculating energy density (E Wh kg^−1^) and power density (W kg^−1^) is as follows:

(2) $$E=\int_{t1}^{t2}IVdt=\frac{1}{2}C\left(V_{max}+V_{min}\right)\left(V_{max}{-V}_{min}\right)$$

(3) $$P=\frac{E}{t}$$

where *V_max_* and *V_min_* are the values of voltage at the end and the start of the discharge process.

## 3. Results and Discussion

**Figure 1:**
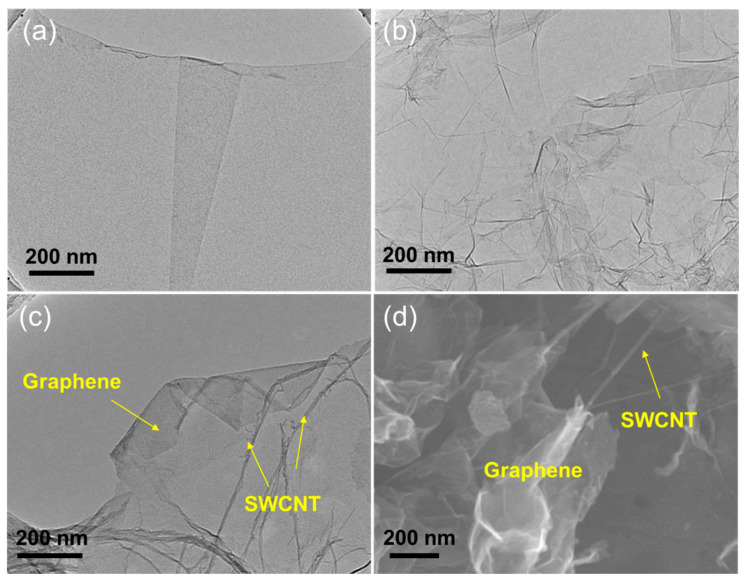
TEM image of (**a**) graphene oxide, (**b**) graphene, (**c**) graphene/SWCNT, and SEM image of (**d**) graphene/SWCNT.

To investigate the morphology of the synthesized graphene/SWCNT and whether the SWCNTs were efficiently inserted into the graphene sheets, TEM and SEM of graphene oxide, graphene, and graphene/SWCNT was carried out, respectively. Figure 1a shows that the graphene oxide microstructure is relatively flat. After chemical reduction, graphene (Figure 1b) exhibits multiple wrinkles and stacking, making it disadvantageous for ion transport in electrochemical reactions. With the addition of single-walled carbon tubes (Figure 1c), it is observed that the graphene state is re-flattened. Moreover, the single-walled carbon tubes, which are very difficult to disperse as nanostructures, are also uniformly distributed among the graphene sheets (Figure 1c,d). This phenomenon occurs because the three-dimensional (3D) network-structured graphene/SWCNT composites reduce the re-stacking.

**Figure 2:**
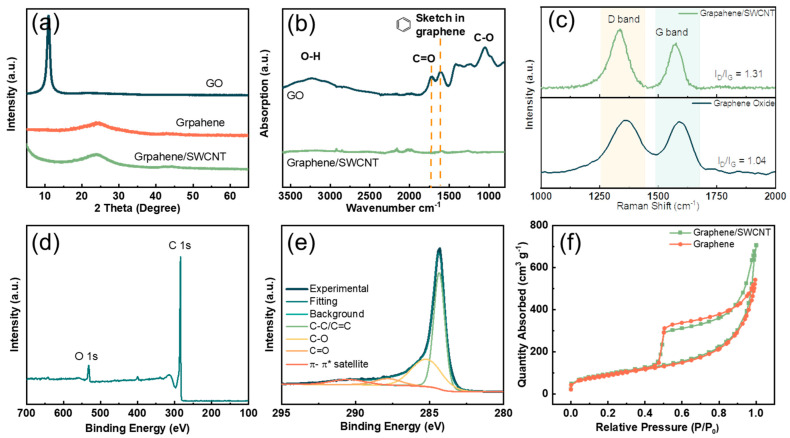
(**a**) XRD patterns of GO, graphene, and graphene/SWCNT; (**b**) FT-IR spectra of GO and graphene/SWCNT; (**c**) Raman spectra of GO and graphene/SWCNT showing the D-band and G-band; (**d**) XPS spectra of graphene/SWCNT. (**e**) analysis of C1s XPS of graphene/SWCNT. (**f**) Nitrogen adsorption/desorption isotherms of graphene and graphene/SWCNT.

To further explore the microstructure of graphene/SWCNT composites, the XRD results are shown in Figure 2a. Graphene oxide (GO) has the smallest diffraction angle and the smallest half-peak width, and is inferred to have the largest layer spacing. This is due to the preparation of GO using the oxidation method, which has functional groups such as hydroxyl and carboxyl on the surface of graphene oxide. The (002) diffraction peak of graphene/SWCNT is centered at 2θ = 24.2°, which is slightly shifted to a lower angle compared to graphene (2θ = 23.9°), suggesting that the addition of SWCNT slightly increases the layer spacing of graphene. FT-IR spectra (Figure 2b) show that graphene oxide has more functional groups than graphene, such as C-O, C=O, and -OH, on the surface, which is consistent with the XRD results. After co-reduction, there are almost no functional groups on graphene/SWCNT composites, indicating that graphene is sufficiently reduced. In Raman spectroscopy, the G peak at ~1580 cm^−1^ represents the material’s carbonization degree, and the D peak at ~1350 cm^−1^ represents the defects and disordered structure in the hexagonal lattice of graphene. The D and G peaks’ intensity ratio (ID/IG) can usually be used to assess the degree of defects in carbon materials [25]. Figure 2c shows the Raman spectra of GO and graphene/SWCNT. Due to the removal of oxygen-containing functional groups, graphene restores the structure of the hexagonal lattice [26]. Thus, the G peak moves from 1592 cm^−1^ for GO to 1573 cm^−1^ for graphene/SWCNT. Compared to the I_D_/I_G_ value of 1.03 for GO, the I_D_/I_G_ ratio of reduced graphene/SWCNT increases to 1.31, indicating that more lattice defects are introduced during the reduction process. Although the reduced graphene restores the conjugated sp^2^ carbon structure, some vacancies are left at the positions bound to the carbon atoms with the removal of oxygen-containing functional groups. These defects eventually lead to an increase in the I_D_/I_G_ value [27]. 

XPS (Figure 2d) experiments were carried out to verify the functional group content of the graphene/SWCNT surface. The C1s peak was split, and the fitted curve is shown in Figure 2e. At 284.4 eV, significantly firm peaks were attributed to the graphite carbon (C-C/C=C bond). The weak peaks at 285.2 eV and 288.0 eV, 290.7 eV are due to C-O and C=O, π-π satellite bonds, respectively [28,29,30]. The π-π satellite bonds were caused by the shake-up process of the pi electrons of graphene and CNTs. The specific relative contents of each chemical bond are shown in Figure S1. The relative content of C-C/C=C bonds is as high as 94.01%. The other carbon–oxygen bonding contents were calculated to be 4.43% C-O and 1.56% C=O. The high content of carbon-carbon bonds implies that hydrazine effectively reduces GO to graphene. To characterize the variation in the specific surface area after SWCNT incorporation, nitrogen adsorption/desorption isotherms were obtained, and the results are shown in Figure 2f. According to the IUPAC classification, all the curves showed typical type IV isotherms [31], indicating the presence of micropores and mesopores. The surface areas of graphene/CNT and graphene were 512 m^2^ g^−1^ and 467 m^2^ g^−1^, respectively. It is evident from the shape of the hysteresis loop that under high pressure, graphene/SWCNT has a more mesoporous structure compared to graphene, indicating that the carbon nanotubes are effectively incorporated between the graphene sheets, preventing the graphene sheets from re-stacking. The graphene/SWCNT composite exhibits a larger specific surface area, which is more beneficial for absorbing electrolytes and obtaining high-capacity cathode materials.

LICs, as asymmetric electrochemical devices, have different charging and discharging mechanisms for the cathode and anode. LICs’ full cell capacity is related to cathode and anode capacity by the following equation, where C is the capacity.

(4) $$\frac{1}{Ccell}=\frac{1}{Ccathode}+\frac{1}{Canode}$$

Since the anode is pre-lithiated and has a capacity much larger than the cathode, the capacity of the LIC full cell depends on the cathode [32]. Therefore, we first investigated the non-Faradaic process of the cathode, which works similarly to EDLC.

**Figure 3:**
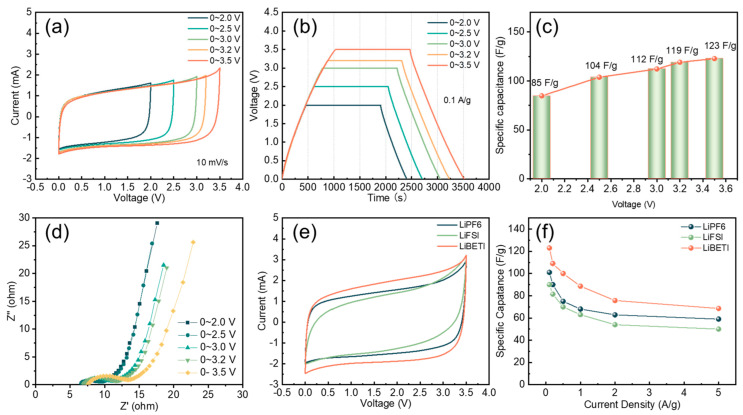
Cathode electrochemical EDLC performance of graphene/SWCNT materials in different electrolytes: (**a**) cyclic voltammograms (CV) curves in LiBETI with scanning rate 10 mV/s, (**b**) galvanostatic charge–discharge curves under different voltages in LiBETI, with 0.1 A/g (**c**) specific capacitance of graphene/SWCNT in LiBETI with different voltage, (**d**) EIS performance of the Nyquist plot under different voltage in LiBETI, (**e**) CV curves, and (**f**) rate performance in LiPF_6_, LiBETI, and LiFSI at 3.5 V.

To evaluate the electrochemical behavior of graphene/SWCNT composites as cathodes with the LiBETI, LiFSI, and LiPF_6_ electrolyte, symmetric capacitor cells were assembled (Figure S2). The cyclic voltammograms (CV) of the LiBETI capacitor within the potential window of 0 to 2.0, 2.5, 3.0, 3.2, and 3.5 V are shown in Figure 3a. The CV curves are nearly rectangular. The galvanostatic current charge/discharge curve is shown in Figure 3b, and the specific capacitance at each voltage is calculated from the discharge curve, as shown in Figure 3c. With increasing voltage, the specific capacitance of graphene/SWCNT can reach 123 F/g at 3.5 V. At a high voltage of 3.5 V, the charge/discharge curves remain parallel to those at low potentials, indicating that the electrolyte exhibits stable electrochemical performance at high voltages. In addition, the EIS (Figure 3d) was tested after charging and discharging at various voltages. The series resistance (Rs), defined as the internal resistance of the electrode in the electrolyte, is typically taken from the high-frequency region of the Nyquist plot. The charge transfer resistance (Rct), measured between the electrode and electrolyte, is taken from the diameter in the Nyquist plot [33,34] with the voltage increasing from 2.0 V to 3.5 V, The Rs is 6.6 Ω, 6.6 Ω, 6.7 Ω, 6.8 Ω, 7.3 Ω. To explore the difference in electrochemical behavior between LiBETI and the commonly used LIC electrolyte LiPF_6_, LiFSI, the CV curves with scan rate 10 mV/s at 3.5 V are shown in Figure 3e. At a high voltage of 3.5 V, the CV curves of both LiPF_6_ and LiFSI cannot maintain a parallelogram, attributed to the polarization reaction of the electrolyte [35]. Therefore, LiBETI electrolytes can be used at higher voltages compared to LiPF_6_ and LiFSI. Compared to the rate performance of the three electrolytes shown in Figure 3f, it is proven that LiBETI has a larger specific capacitance and better rate performance.

In addition, the outstanding capacity (compared with the commercial active carbon capacity of 100 F/g at 3.0 V) of graphene/SWCNT composites in EDLC is attributed to their unique three-dimensional network structure. The main advantages of the composites are as follows: (1) the graphene/SWCNT composites have a large specific surface area and a rich mesoporous structure that can effectively absorb the electrolyte; (2) the use of single-walled carbon nanotubes as spacers prevents graphene from re-stacking; and (3) the integration of carbon tubes enhances the electrical and thermal conductivity of the composites. As discussed by Yuan et al., the electrical conductivity of SWCNT in its axial direction is very high, which makes SWCNT an excellent conductive binder in composites [19,36,37].

**Figure 4:**
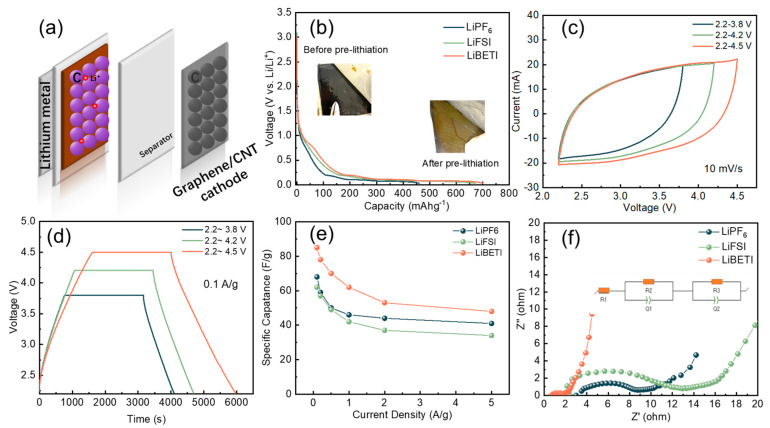
(**a**) Schematic diagram of LIC full cell structure. (**b**) Anode pre-lithiation discharge curves in different electrolytes to 0.08 V at 20 mA g^−1^. The inset shows the anode comparison before and after pre-lithiation in LiBETI electrolyte. The LIC full cell electrochemical performance: (**c**) CV curves in LiBETI with scanning rate 10 mV/s, (**d**) galvanostatic charge–discharge curves under different voltage in LiBETI, with 0.1 A/g. (**e**) Rate performance in LiPF_6_, LiBETI, and LiFSI at 2.2~4.5 V. (**f**) EIS performance of Nyquist curves in LiPF_6_, LiBETI, and LiFSI, and the inset shows the fitted circuit in LiBETI.

A three-electrode LIC full cell was assembled to characterize the electrochemical performance. The cell structure was shown in Figure 4a. The mass ratio between the cathode and anode is 7:3. Lithium foil was used as the reference electrode, graphene/SWCNT as the cathode, and graphite (type P5B, pitch coated graphite) as the anode. The discharge curves for anode pre-lithiation are shown in Figure 4b. The graphite anode exhibits a higher capacity in LiBETI at the same pre-lithiation current and voltage (LiPF_6_: 452 mAh/g, LiFSI: 668 mAh/g, LiBETI: 700 mAh/g). In the LiBETI electrolyte, the electrode color change before and after pre-lithiation is shown in the illustration, and the electrode changes from black to brownish-yellow, which indicates that after pre-lithiation, the solid electrolyte interface (SEI) film is generated, and the Li ions are intercalated in the graphite [38,39]. To prevent anode over-discharge [40], the LIC has a discharge cutoff potential of 2.2 V. We also set up experiments at different measurement potential with 2.2~3.8 V (commercial LICs potential 2.2~3.8 V), 2.2~4.2 V, 2.2~4.5 V. First, in LiBETI, the shape of the CV curves (Figure 4c) is well maintained at different voltages for the LIC full cell, indicating the superior reversible property. As shown by the charge and discharge curves in Figure 4d, the specific capacitance of LIC full cell with 3.8 V, 4.2 V, and 4.5 V at 0.1 A g^−1^ current density is calculated to 58 F g^−1^, 61 F g^−1^, and 85 F g^−1^, respectively. The cathode-specific capacitance was calculated using the full cell, (Equation (4)), and it was found that the cathode-specific capacitance was 83 F/g, 88 F/g, 121 F/g, at 4.5 V in LiPF_6_, LiFSI, and LiBETI, respectively. This is consistent with its EDLC specific capacitance at 3.5 V. The rate performance is shown in Figure 4e. The specific values are shown in Table S1. It was found that in LIC full-cell devices, LiBETI electrolytes show the highest specific capacitance and better rate performance.

To investigate the resistance of LIC full cells in LiPF_6_, LiFSI, and LiBETI, Figure 4f compares Nyquist curves. The slope LiBETI > LiPF_6_ > LiFSI in the low-frequency region indicates that LiBETI has the fastest ion diffusion rate and more optimal capacitive behavior. In the high-frequency part, the arc radius in high-frequency area LiBETI < LiPF6 < LiFSI, indicating that the electrode has lower charge transfer resistance and a faster ion transfer rate in the LiBETI electrolyte. The equivalent circuit was fitted using EC-Lab; the included circuit is shown in the inset of Figure 4f, and the fitting result is shown in Figure S3.

**Figure 5:**
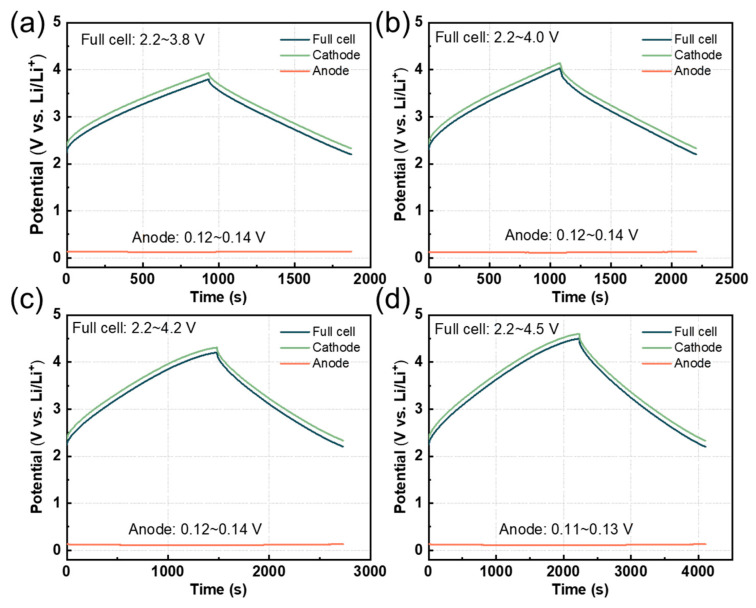
Three-electrode LIC charge/discharge curve with lithium as a reference electrode. Cathode, anode, and LIC full-cell potential at a current density of 0.1 A g^−1^ with different voltages (**a**) 3.8 V; (**b**) 4.0 V; (**c**) 4.2 V; and (**d**) 4.5 V.

To investigate the electrochemical behavior of the cathode and anode further, a three-electrode system was used to explore the potential changes in the cathode and anode. As shown in Figure 5, the potential of the anode remained constant as the charging voltage increased during the charging and discharging process. When the voltage was increased to 4.5 V, the anode potential was close to that achieved when charging to 3.8 V, with a drop of only 0.01 V. From this phenomenon, it can be inferred that the generation of an anode SEI film was sufficiently stable.

**Figure 6:**
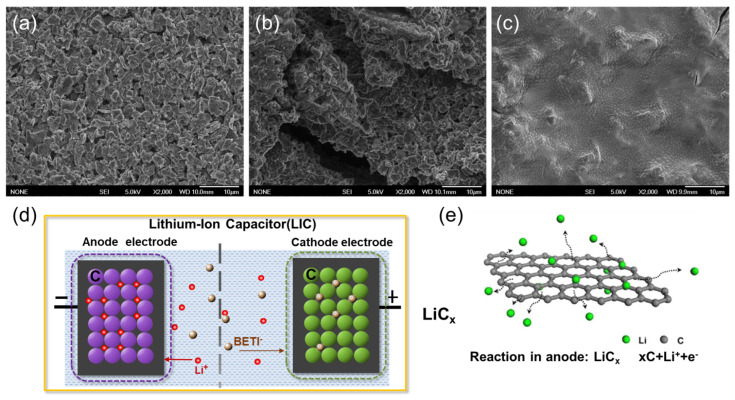
SEM images of graphite anode film after 50 cycles (**a**) with LiPF_6_, (**b**) with LiFSI, (**c**) with LiBETI, (**d**) charge and discharge mechanism schematic in LIC, and (**e**) schematic diagram of graphite anode structure after pre-lithiation.

To confirm whether the anode SEI film was stable, after 50 charging and discharging cycles at 2.2–4.5 V voltage, we performed an SEM test on the anode electrode film to confirm the surface morphology of the electrode film. The anode in the LiPF_6_ electrolyte (Figure 6a) was arranged evenly and the graphite block with the size about 3–6 μm. However, compared with the graphite anode in LiPF_6_, in LiFSI, the surface of the anode graphite material was not flat, and cracks were observed in Figure 6b) [41]; the size of the block did not change significantly, indicating that the anode deteriorated in the LiFSI electrolyte as a result of high voltage cycling. Significantly, a dense SEI film was generated on the anode surface in the LiBETI electrolyte, which can be observed in Figure 6c. It was also verified that the LiBETI electrolyte can help to form a stable SEI film during the anode prelithiation process. As shown in Figure 6d, the LIC full cell presents a typical hybrid charging and discharging mechanism that combines battery and capacitor energy storage. During charging, anions (BETI^−^) in the electrolyte are adsorbed to the cathode due to electrostatic interaction, and Li^+^ intercalates into the anode. When the cell is discharged from the maximum voltage, the BETI^−^ ions desorb from the cathode and the Li^+^ ions de-intercalate from the anode into the electrolyte. In LIC, lithium storage in graphite is achieved through intercalation between graphite layers, and the LiC_x_ structure is shown in Figure 6e. 

**Figure 7:**
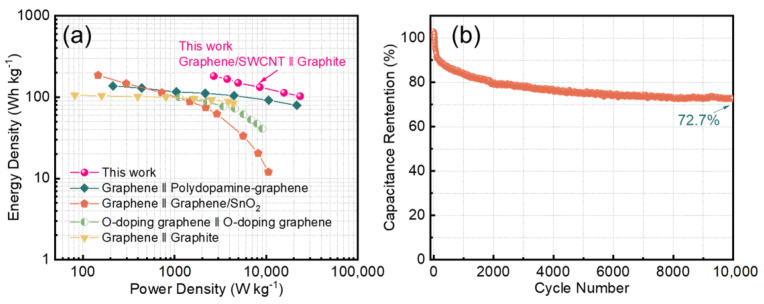
(**a**) Ragone plot of graphene/SWCNT LIC with LiBETI in comparison with previously reported LICs, (**b**) cycle performance of graphene/SWCNT LIC with LiBETI in 2.2~4.5 V.

The power density and energy density of the graphene/SWCNT LIC are shown in Figure 7a in comparison with the previously reported LICs. With the potential of 2.2–4.5 V, it can be revealed that the graphene/SWCNT LIC achieves a high energy density of 182.6 Wh kg^−1^ at a power density of 2678.0 W kg^−1^. Even at a high-power density of 23,437.1 W kg^−1^, the graphene/SWCNT LIC maintains a high energy density of 102.7 Wh kg^−1^, which exhibits excellent rate performance. Compared with others’ work, for example, the previous work Graphene ‖ Graphene/SnO_2_ LIC shows a density of 186.0 Wh kg^−1^ at a power density of 146.3 W kg^−1^. It offers a similar energy density to us, but the power density is much lower. The specific energy density and power density values of other works are in Table S2 [42,43,44,45]. Moreover, the cycling performance of the assembled graphene/SWCNT LIC was also investigated (Figure 7b). It showed excellent cycling stability at high voltage from 2.2 to 4.5 V with a current density of 2 A g^−1^ and exhibited a high-capacity ratio of 72.7% after 10,000 cycles, which was attributed to the stabilized anode SEI film that kept the anode potential constant and stabilized the LIC voltage window at high potential. The assembled high-voltage, high-energy-density LICs show broad prospects for practical applications.

## 4. Conclusions

In conclusion, we synthesized graphene/SWCNT 3D structure nanocomposites, in which SWCNT effectively prevents graphene re-stacking and provides a large specific surface area, which offers additional active sites for ion adsorption. The three-electrode system was explored to investigate LiBETI as an electrolyte with high conductivity and excellent electrochemical and mechanical stability. As a result, the asymmetric LIC device assembled using graphene/SWCNT as the cathode and pre-lithiated graphite as the anode has a specific capacitance of up to 85 F g^−1^ and maintains a capacity retention of up to 72% after 10,000 cycles. In addition, it exhibits a maximum energy density of 182.6 Wh kg^−1^ at a power density of 2678.0 W kg^−1^. Significantly, this work opens up a new avenue for the practical application of low-cost, advanced carbon materials for next-generation energy-storage technologies.

## Data Availability

Data are contained within the article or Supplementary Materials.

## Supplementary Materials

The following supporting information can be downloaded at https://www.mdpi.com/article/10.3390/nano14010045/s1, Figure S1: The specific relative contents of each chemical bond of graphene/SWCNT. Figure S2: Schematic diagram of symmetrical capacitor structure. Table S1: Specific capacitance of LIC in different electrolytes with different current densities. Figure S3: LIC fitting equivalent circuit in LiBETI electrolyte. Table S2: The performance of the previously reported LICs comparison to Graphene/SWCNT LIC.
