# Supplementary material for: Achieving High-Energy-Density Graphene/Single-Walled Carbon Nanotube Lithium-Ion Capacitors from Organic-Based Electrolytes
Source: Nanomaterials (Basel). 2023 Dec 22;14(1):45. doi: 10.3390/nano14010045 (PMC10780324; doi:10.3390/nano14010045)
Supplement: Supplementary file 1 [file nanomaterials-14-00045-s001.zip › nanomaterials-2759259-supplementary.pdf]

nanomaterials

Support Information

Achieving High-Energy-Density Graphene/Single-Walled Carbon Nanotube Lithium-Ion Capacitors from Organic-Based Electrolytes

Hang Yin [1,2], Jie Tang [1,2,*], Kun Zhang [1], Shiqi Lin [1], Guangxu Xu [1,2] and Lu-Chang Qin [3]

1 National Institute for Materials Science, 1-2-1 Sengen, Tsukuba 305-0047, Ibaraki, Japan; yin.hang@nims.go.jp (H.Y.); zhang.kun@nims.go.jp (K.Z.); lin.shiqi@nims.go.jp (S.L.); xu.guangxu@nims.go.jp (G.X.)
2 Graduate School of Pure and Applied Sciences, University of Tsukuba, 1-1-1 Tennodai, Tsukuba 305-0006, Ibaraki, Japan
3 Department of Physics and Astronomy, University of North Carolina at Chapel Hill, Chapel Hill, NC 27599-3255, USA; lcqin@email.unc.edu
* Correspondence: tang.jie@nims.go.jp; Tel.: +81-02-98592728

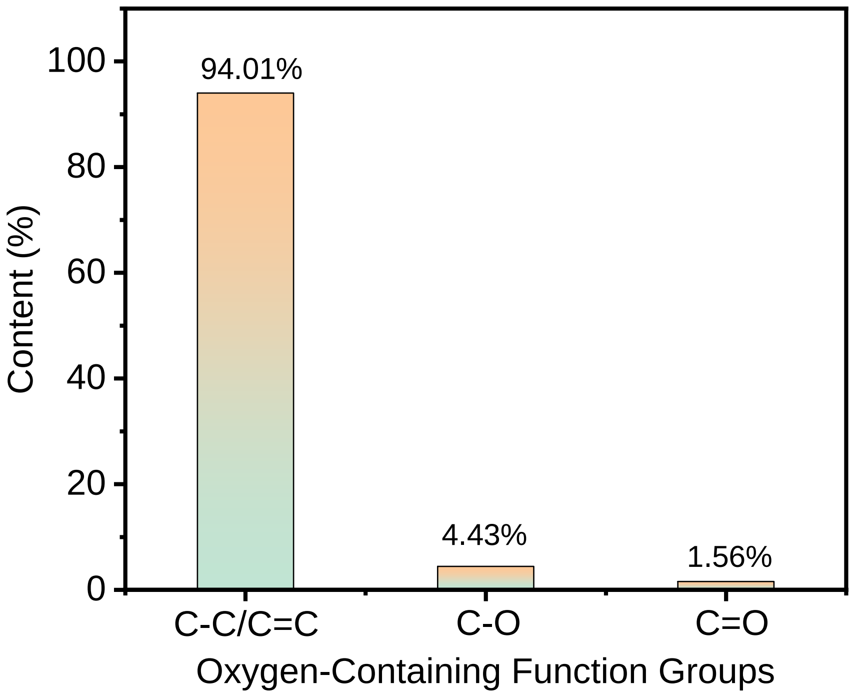

Figure S1. The specific relative contents of each chemical bond of graphene/SWCNT.

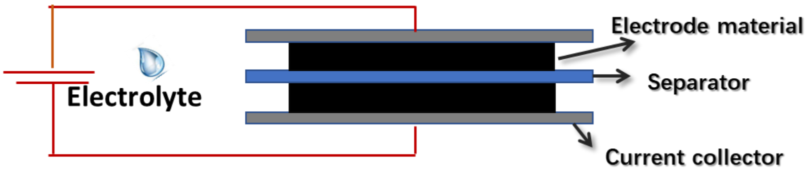

Figure S2. Schematic diagram of symmetrical capacitor structure.

Table S1. Specific capacitance of LIC in different electrolytes with different current densities.

Current Density (A/g)	$LiPF_6$ (F/g)	LiFSI (F/g)	LiBETI (F/g)
0.1	68	62	85
0.2	59	57	78
0.5	50	49	70
1.0	46	42	62
2.0	44	37	53
5.0	41	34	48

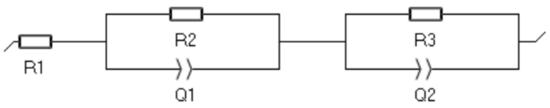

Figure S3. LIC fitting equivalent circuit in LiBETI electrolyte.

Equivalent circuit:
R1 + R2/Q1 + R3/Q2
R1 = 7.199e–45 Ohm
R2 = 17.64e−15 Ohm
Q1 = 59.44e−6 F. s^ (a − 1)
a1 = 1
R3 = 1.24 Ohm
Q2 = 10.79e-21 F. s^ (a − 1)
a2 = 0.990 5

Table S2. The performance of the previously reported LICs comparison to Graphene/SWCNT LIC.

LICs	Power Density (W/kg)	Energy Density (Wh/kg)
Graphene ‖ Polydopamine-graphene	212.7	137.1
Graphene ‖ Graphene/SnO_2	146.4	186.0
O-doping graphene ‖ O-doping graphene	1126.4	99.4
Graphene ‖ Graphite	4395.3	84.4
Graphene/SWCNT ‖ Graphite (This work)	2678.5	181.9
